# Genomic Resources for Imperiled Caribbean Reef‐Forming Corals (Hexacorallia: Scleractinia): Complete Mitochondrial Genomes of *Dichocoenia stokesii*, *Diploria labyrinthiformis*, *Oculina patagonica*, and *Stephanocoenia intersepta*

**DOI:** 10.1002/ece3.72967

**Published:** 2026-01-30

**Authors:** Katrina Zabransky, William Vuong, Stephanie M. Rosales, J. Antonio Baeza

**Affiliations:** ^1^ Department of Biological Sciences Clemson University Clemson South Carolina USA; ^2^ Cooperative Institute for Marine and Atmospheric Studies The University of Miami Miami Florida USA; ^3^ Atlantic Oceanographic and Meteorological Laboratory National Oceanic and Atmospheric Administration Miami Florida USA; ^4^ Smithsonian Marine Station at Fort Pierce Smithsonian Institution Fort Pierce Florida USA; ^5^ Departamento de Biología Marina Universidad Catolica del Norte Coquimbo Chile

## Abstract

Coral reefs provide a wide variety of services essential to both marine ecosystems and human societies yet reef‐forming corals are currently facing a multitude of global and local environmental stressors. Long‐term monitoring of reef‐forming corals is of utmost importance for understanding the response of declining coral populations to environmental insult, and for their restoration. This study is a part of a greater long‐term goal that aims at generating genomic resources for imperiled corals in the greater Caribbean basin. To support the future monitoring of coral reefs using non‐intrusive eDNA strategies, we have sequenced for the first time the mitochondrial genome of four species of corals, three of them inhabiting the greater Caribbean basin: *Dichocoenia stokesii*, *Diploria labyrinthiformis* , *Oculina patagonica* , and *Stephanocoenia intersepta* and examined their phylogenetic placement. The mitochondrial genomes of *Dicho. stokesii*, *Diplo. labyrinthiformis*, *O. patagonica* , and *S. intersepta* are 17,171, 16,905, 14,856, and 19,461 bp in length respectively. All four studied coral mitochondrial genomes contain 13 protein coding genes (PCGs), 2 transfer RNA genes (tRNA), and 2 ribosomal RNA genes (rrnS and rrnL). In all four studied mitochondrial genomes, a long group I intron bisected the *nad5* gene. Additionally, *S. intersepta* has a second group I intron 942 bp in length that bisected the *cox1* gene, overlapping the *nad5* group I intron. The rrL gene in the mitochondrial genome of *S. intersepta* is also bisected by a 7259 bp intron. The phylogenetic position of the species with newly assembled mitochondrial genomes was examined using a Maximum Likelihood phylogenetic analysis based on PCGs. These newly developed genomic resources (mitochondrial genomes) will support eDNA biomonitoring of reef‐forming coral inhabiting the greater Caribbean basin.

## Graphical abstract

Mitochondrial genomes of imperiled Caribbean corals are sequenced. These genomic resources will support conservation initiatives.

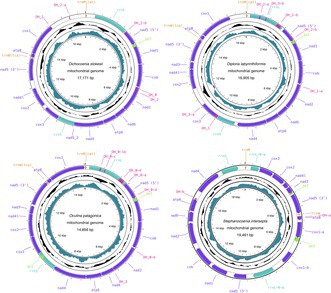

## 1 Introduction

Coral reefs and corals, both hard (Hexacorallia: Scleractinia) and soft (Octocorallia), which comprise the structural foundation of these reefs, provide a wide variety of services essential to both marine ecosystems and human societies (Moberg and Folke 1999). Coral reefs, although occupying less than 0.1% of the ocean's floor (Spalding et al. 2001), provide habitat for an estimated 830,000 (95% confidence limits: 550,000–1,330,000) species of both invertebrates and vertebrates (Fisher et al. 2015). Subsequently, coral reefs maintain healthy food webs and ecosystem stability. Furthermore, together with mangrove forests, coral reefs, and especially shallow coral reefs, act as natural barriers that dissipate wave energy, reducing wave height to protect coastlines from baseline rates of erosion and storm and hurricane damage, which are crucial for coastal communities and decrease the need for artificial (man‐made) defenses (Ferrario et al. 2014). From an economic standpoint, coral reefs support fisheries and tourism, contributing billions of dollars to global economies (Moberg and Folke 1999; Kittinger et al. 2012) and coral, sea anemones, sponges, and tunicates, among other coral reef constituents, produce a wide array of bioactive chemical compounds exploited by pharmaceutical industries (Carté 1996).

Worldwide, reef‐building corals are currently facing a multitude of global and local environmental stressors contributing to the decline of their populations (Kleypas et al. 1999; Pandolfi et al. 2011). Among the most important global stressors, rising water temperatures and acidification are causing episodic coral bleaching and a decrease in the ability of corals to maintain and form their calcium carbonate skeletons (Hoegh‐Guldberg et al. 2007; Baker et al. 2008), respectively. Overall, despite their resilience, global change threatens the functional stability and diversity of coral reefs. In turn, local stressors include, among others, pollution from land‐based runoff, which introduces excess nutrients and toxins encouraging excessive algal growth (Dubinsky and Stambler 1996), overfishing, which can lead to loss of keystone species and food web predators (Roberts 1995), and reduces herbivore populations that control algal growth in coral reefs (Rasher et al. 2012), and habitat destruction and alteration with decreased functional diversity, driven, in great part, by destructive fishing practices (Roberts 1995) and coastal development (Bozec et al. 2008).

In the Greater Caribbean Sea, coral reefs are deteriorating, and mortality of reef‐forming corals is similar to or greater than that reported worldwide (Hughes 1994). The Greater Caribbean basin is home to approximately 60–70 species of scleractinian corals (Cramer et al. 2020) and at least 61 soft corals (Octocorallia), contributing to the reef's overall biodiversity (Sanchez and Wirshing 2005). One of the most challenging conservation goals in the greater Caribbean basin and beyond is the restoration of their degraded coral reefs (Bayraktarov et al. 2020). Long‐term monitoring is of utmost importance for understanding the response of declining coral populations to environmental insult, and subsequently, for the efficient development and implementation of conservation measures, including their restoration (Obura et al. 2019). Unfortunately, in situ monitoring strategies and collection of biological samples for genomic research are often difficult to implement due to logistical challenges and ethical concerns, particularly in threatened and endangered coral species with predicted short‐ and/or long‐term decaying population trajectories (Hedley et al. 2016). Such limitations impact conservation efforts and traditional in situ and “invasive” bioprospecting and bio‐monitoring strategies that can disturb and/or stress corals already facing major anthropogenic impacts (Hughes 1994). These strategies may need to be replaced by other non‐invasive strategies when needed. Fortunately, non‐intrusive environmental DNA (eDNA) has materialized during the last years as a reliable substitute or complement to invasive and/or intrusive sampling strategies for the biomonitoring of imperiled marine species, including corals (Beng and Corlett 2020; Didaskalou et al. 2022). However, there is a lack of genomic resources for Caribbean corals. The number of species with an assembled nuclear and/or mitochondrial genome is limited but rapidly increasing during the last years (Selwyn and Vollmer 2023 and references therein) though the great majority of corals in this region do not have major genetic and genomic resources. These lack of genomic resources (i.e., reference mitochondrial genomes) is constraining the development of non‐intrusive protocols for the accurate biomonitoring of coral reefs.

This study is a part of a greater long‐term goal that aims at generating genomic resources for imperiled corals in the greater Caribbean basin (see Tucker et al. 2023; Baeza 2025; Baeza and Rosales 2025). To support the future monitoring of coral reefs using non‐intrusive eDNA strategies, we have sequenced for the first time the mitochondrial genome of 4 species of corals, 3 of them from the greater Caribbean basin: *Dichocoenia stokesii*, *Diploria labyrinthiformis* , *Oculina patagonica* , and *Stephanocoenia intersepta* . The 2025 IUCN Red List of Threatened Species classifies *Dicho. stokesii* as “vulnerable,” *Diplo. labyrinthiformis* as “critically endangered,” *O. patagonica* as “least concern,” and *S. intersepta* as “near threatened” (IUCN 2025). Following suggestions in Baeza (2022), we have analyzed their mitochondrial genomes in detail; we have estimated nucleotide composition, codon usage in PCGs, secondary structures of the tRNA genes, and repetitive elements in the control region. Lastly, we have explored the phylogenetic placement of the different studied species using the phylogenomic informativeness provided by translated protein coding genes.

## 2 Materials and Methods

We collected and sequenced specimens belonging to three different Caribbean corals: *Dichocoenia stokesii*, *Diploria labyrinthiformis* , and *Stephanocoenia intersepta* following the protocol of Rosales et al. (2020). In short, while SCUBA diving during June 2018 (06/29/2018), from the shallow water patch reefs (Washerwoman Reef) within the Florida Keys National Marine Sanctuary (FKNMS), coral samples were collected by scraping 10‐ml plastic blunt tip syringes on a small area of the coral surface, and simultaneously pulling the syringe plunger until the syringe was full of a slurry consisting of coral tissue and mucus (Rosales et al. 2020). Coral species were visually identified by a trained coral biologist (Dr. Lindsay Huebner, Fish and Wildlife Research Institute [FWRI]). After collections, on a boat, coral slurry samples were transferred from the syringes to 15‐mL plastic tubes that were placed in a dark cooler on ice for transport back to a dry laboratory (Rosales et al. 2020). There, tubes with coral slurry were flash‐frozen in a liquid nitrogen dewar and transferred to a −80°C freezer at Mote Marine Laboratory in Summerland Key, FL for storage until genomic DNA (gDNA) extractions that were performed with the DNeasy PowerSoil Kits (QIAGEN, Germantown, MD, United States) following the manufacturer's instructions. Next, a paired‐end (PE, 150 bp) shotgun library was constructed by MR DNA1 (Shallowater, TX, United States) and sequenced on an Illumina HiSeq platform (Sacramento, CA) following the manufacturer's instructions. Library preparation was conducted using the Roche KAPA HyperPrep Kit, and final libraries were quantified by qPCR. All libraries met quality control standards and were sequenced on an Illumina HiSeq 4000 platform generating 150 bp paired‐end reads. A total of 53,710,335, 46,234,365 and 44,459,687 reads in FASTQ format were generated and used for assembling the mitochondrial genomes of *Dicho. stokesii*, *Diplo. labyrinthiformis*, and *S. intersepta* , respectively. Lastly, we mined NCBI's GenBank for additional short read Illumina reads and assembled the mitochondrial genome of the invasive *O. patagonica* (Bioproject: PRJNA661426, Biosample: SAMN16057441, SRA: SRR28110921). Details on specimen collection, gDNA extraction, and sequencing can be found in Martin‐Cuadrado et al. (2024). The totality of the reads available in GenBank (*n* = 31,952,557) were used for assembling the mitochondrial genome of *O. patagonica* .

### 2.1 De‐Novo Assembly of New Mitochondrial Genomes

The pipeline GetOrganelle v1.2.3 (Jin et al. 2020) was used to assemble the mitochondrial genomes of the studied species. As a “seed,” we used the previously published mitochondrial genome from the closely related coral *Galaxea fascicularis* (NC_029696) to assemble the mitochondrial genomes of *Dichocoenia stokesii*, *Diploria labyrinthiformis* , *Oculina patagonica* , and *Stephanocoenia intersepta* . All GetOrganelle runs used k‐mer sizes equal to 21, 55, 85, and 115 (Jin et al. 2020).

### 2.2 Annotation of the Newly Assembled Mitochondrial Genomes

The assembled mitochondrial genomes of *Dicho. stokesii*, *Diplo. labyrinthiformis*, *O. patagonica* , and *S. intersepta* were annotated using the invertebrate genetic code in the pipeline MITOS2 (Bernt et al. 2013) as implemented in the platform Galaxy EU (https://usegalaxy.eu/—The Galaxy Community 2024). PCG annotations were manually curated using the web server Expasy translate (https://web.expasy.org/—Artimo et al. 2012) and the software Mega X (Kumar et al. 2018). Once curated, the mitochondrial genomes were visualized with the web server Proskee (https://proksee.ca/—Stothard and Wishart 2004).

The nucleotide composition of each studied mitochondrial genome was estimated using the software Mega X (Kumar et al. 2018). Codon usage of the 13 PCGs present in each mitochondrial genome was calculated using the website Sequence Manipulation Suite: Codon Usage (https://www.bioinformatics.org/sms2/codon_usage.html—Stothard 2000). Also, RSCU (Relative Synonymous Codon Usage) for all concatenated PCGs in each studied mitochondrial genome was estimated and visualized using the EZCodon tool in the web server EZmito webpage (http://ezmito.unisi.it/ezcodon—Cucini et al. 2021).

The secondary structure of each tRNA in the mitochondrial genomes of *Dicho. stokesii*, *Diplo. labyrinthiformis*, *O. patagonica* , and *S. intersepta* was predicted using the default parameters in the program MIFTI (Jühling et al. 2012) available through MITOS2 in Galaxy EU (https://usegalaxy.eu/—Bernt et al. 2013; The Galaxy Community 2024) and each tRNA secondary structure was visualized using the web server Forna (http://rna.tbi.univie.ac.at/forna—Kerpedjiev et al. 2015).

Relatively long (> 500 bp) non‐coding regions present in the *Dicho. stokesii*, *Diplo. labyrinthiformis*, and *S. intersepta* mitochondrial genomes were analyzed in detail. *O. patagonica* mitochondrial genome did not have a non‐coding region greater than 500 bp. In *O. patagonica* the longest non‐coding region was 109 bp long, and was analyzed in detail. The presence/absence of microsatellites (with motifs ranging in length between 2 and 5 bp) was determined in the website BioPHP Microsatellite Repeats Finder using the default parameters (http://insilico.ehu.es/mini_tools/microsatellites/—Bikandi et al. 2004). The control regions were also analyzed for short tandem repeats (with motif length > 5 bp) using the web server Tandem Repeat Finder with the Alignment Parameters option (match = 2, mismatch = 3, indels = 5) (https://tandem.bu.edu/trf/trf.basic.submit.html—Benson 1999). Lastly, to visualize and predict the optimal minimum free energy secondary structure of the non‐coding regions, the web server RNAfold (http://rna.tbi.univie.ac.at/cgi‐bin/RNAWebSuite/RNAfold.cgi—Gruber et al. 2008) was used with default parameters putting attention to existence of “hairpin” structures.

### 2.3 Phylomitogenomics of Scleractinian Corals

To determine the phylogenetic position of the studied species in the subclass Hexacorallia, the 4 newly assembled mitochondrial genomes, along with all annotated mitochondrial genomes within the subclass Hexacorallia available in NCBI's GenBank (*n* = 164), were included for phylogenetic inference using maximum likelihood (ML). The phylogenetic analysis was conducted using plugin tools in the software PhyloSuite (Zhang et al. 2020). First, all protein coding gene nucleotide sequences were extracted and aligned with the program MAFFT (Katoh et al. 2002). The software TrimAl (Capella‐Gutiérrez et al. 2009) was then used to remove poorly aligned regions, if any, in each aligned set of PCG sequences. ModelFinder (Kalyaanamoorthy et al. 2017) was used to determine the best‐fitting model of sequence evolution for the protein‐coding genes that then were concatenated to conduct a ML phylogenetic analysis with the program IQ‐TREE version 1.6.10 using default parameters (Nguyen et al. 2015). The robustness of the ML tree topology was assessed by 1000 bootstrap iterations. Finally, the phylogenetic tree was depicted using the web server Interactive Tree of Life (iTOL v5) (Letunic and Bork 2021). We conducted a second analysis in which nucleotides from each of the 13 PCGs were translated to amino acids using genetic code 5 with the program MAFFT (Katoh et al. 2002). The pipeline employed in this second analysis was identical to that of the first analysis. We note that tree topology in this second analysis was the same as in the first analysis and we only present the results from the first analysis that used nucleotides.

## 3 Results and Discussion

**FIGURE 1:**
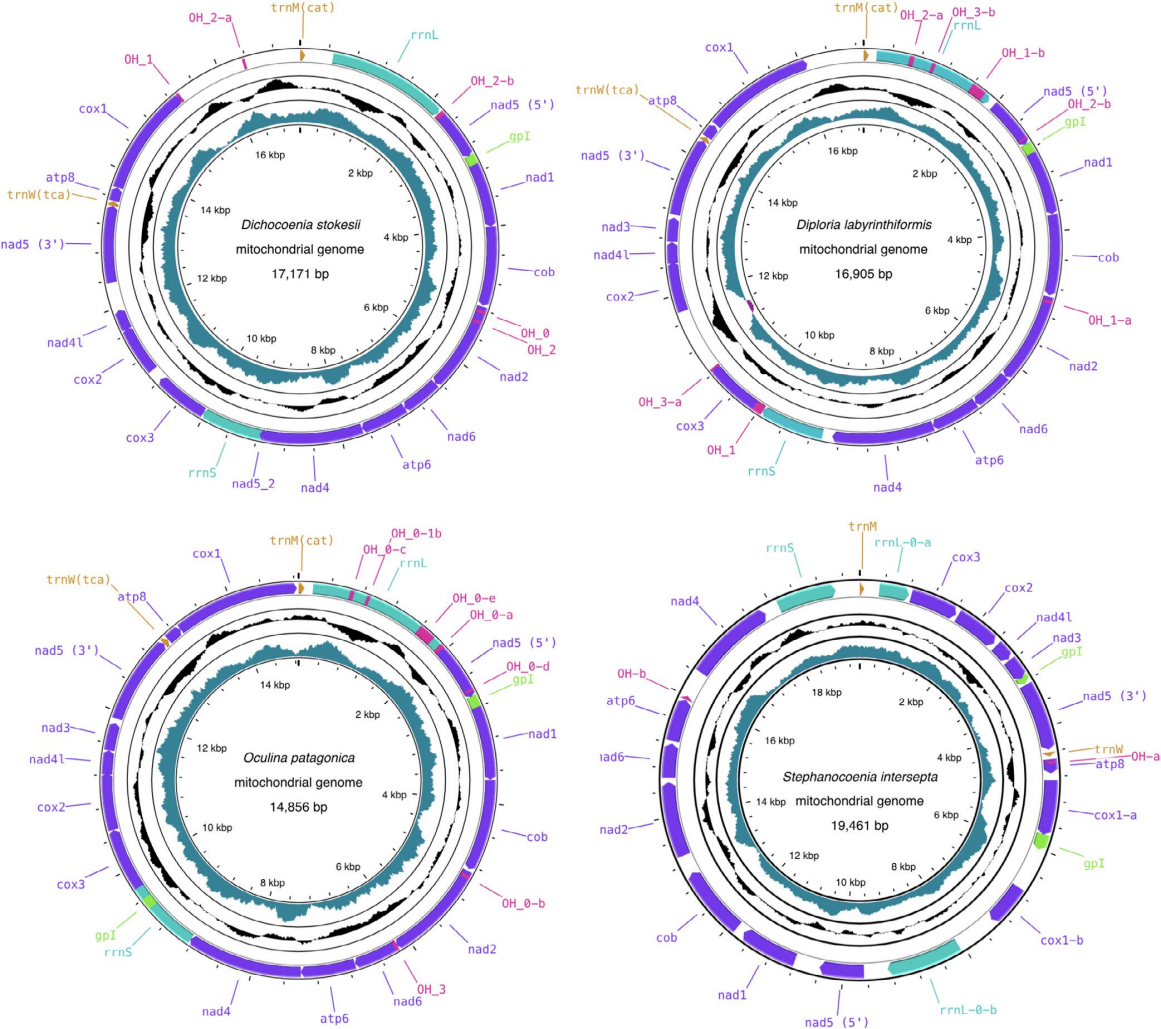
Circular mitochondrial DNA maps of *Dichocoenia stokesii*, *Diploria labyrinthiformis* , *Oculina patagonica* , and *Stephanocoenia intersepta* using the Proskee website (https://proksee.ca/). The two inner circles depict the GC content and the GC skew of the corresponding sequences.

The software GetOrganelle assembled and circularized the mitochondrial genomes of *Dichocoenia stokesii* (GenBank accession number PV487494), *Diploria labyrinthiformis* (GenBank accession number PV487495), *Oculina patagonica* (GenBank accession number PV487496), and *Stephanocoenia intersepta* (GenBank accession number PV423450) with an average coverage of 59x, 188x, 215x, and 939x per nucleotide, respectively. The mitochondrial genomes of *Dicho. stokesii*, *Diplo. labyrinthiformis*, *O. patagonica* , and *S. intersepta* were 17,171, 16,905, 14,856, and 19,461 bp in length respectively. All four studied coral mitochondrial genomes contain 13 protein coding genes (PCGs), 2 transfer RNA genes (tRNA), and 2 ribosomal RNA genes (rrnS and rrnL). All genes for the four mitochondrial genomes were encoded on the heavy strand (Figure 1, Tables S1–S4). The mitochondrial gene order is not identical for the four studied mitochondrial genomes, in line with previous studies that report variable mitochondrial synteny among Scleractinian corals (Tucker et al. 2023).

In all four studied mitochondrial genomes, a long group I intron bisected the *nad5* gene. In *Dicho. stokesii* and *Diplo. labyrinthiformis*, the group I intron was 9425 and 10,443 bp long, respectively and encompassed ten PCGs (*nad1*, *cob*, *nad2*, *nad6*, *atp6*, *nad4*, *cox3*, *cox2*, *nad4l*, *nad3*) and one ribosomal RNA gene (rrnS). In turn, the group I intron in *Oculina patagonica* was 9259 bp in length and encompasses ten PCGs (*nad1*, *cob*, *nad2*, *nad6*, *atp6*, *nad4*, *cox3*, *cox2*, *nad4l*, and *nad3*) and one ribosomal RNA gene (rrnS). Lastly, the group I intron in *S. intersepta* was 5293 bp long and encompassed three protein coding genes (*atp8*, *cox1‐*a, *cox1‐b* [see below]), one ribosomal RNA gene (rrL‐0b), and one tRNA gene (trnM). Additionally, *S. intersepta* has a second group I intron 942 bp in length that bisected the *cox1* gene, overlapping the *nad5* group I intron. Group I introns that bisect *cox1* and *nad5* have been previously identified in various other stony corals (van Oppen et al. 2002; Tucker et al. 2023; Chen, Dai, et al. 2008; Tseng et al. 2005; Fukami and Knowlton 2005) and these group I introns have been hypothesized to be functional and transcribed (van Oppen et al. 2002).

Interestingly, the rrnL gene in the mitochondrial genome of *S. intersepta* is bisected by a 7257 bp intron that encompasses eight PCGs (*cox3*, *cox2*, *nad4l*, *nad3*, *nad5* (3′), *atp8*, *cox1*‐a, *cox1*‐b), one tRNA gene (trnW), two gpI, and one origin of replication. We concluded that this rrnL gene in *S. intersepta* was bisected by an intron and not duplicated because the two DNA sections annotated by Mitos2 as rrnL were not identical or highly similar to each other and considering that when the two mitochondrial rrnL genes in *S. intersepta* were aligned together with three other closely related mitochondrial rrnL genes, the combined length was 1706 bp, which is similar to previously documented coral rrnL gene lengths of 1699 bp (*Dipsastraea favus*—Tong et al. 2019) to 2447 bp ( *Ricordea florida* —Medina et al. 2006). The rrnL gene bisected by an intron is also observed in the mitochondrial genomes of *Letepsammia formosissima* and *L. franki* (Seiblitz et al. 2020).

The overall nucleotide composition of the four studied mitochondrial genomes was A+T rich. The A + T contents for *Dicho. stokesii*, *Diplo. labyrinthiformis*, *O. patagonica* , and *S. intersepta* were 68.2%, 66.5%, 68.2%, and 63.1% respectively (Table 1) and the observed values are similar to the A + T contents reported for other coral mitochondrial genomes, with the largest A + T content observed in *Polycyathus* sp. (70.91%—Lin et al. 2014) and the smallest A + T content reported for *Pavona decussata* (59.27%—Shi et al. 2016).

**TABLE 1:** Nucleotide usage in *Dichocoenia stokesii*, *Diploria labyrinthiformis* , *Oculina patagonica* , and *Stephanocoenia intersepta* complete mitochondrial genomes, rRNA genes, and control regions.

	A (%)	T (%)	G (%)	C (%)	A + T (%)	G + C (%)	Length (bp)
*Dichocoenia stokesii*							
Complete genome	25.5	42.7	19.7	12.0	68.2	31.7	17,171
rrnS	34.2	32.1	22.2	11.5	66.3	33.7	910
rrnL	37.7	32.2	18.6	11.5	69.9	30.1	1723
Control region position: 15,329–17,171	26.7	37.5	21.2	14.6	64.2	35.8	1843
*Diploria labyrinthiformis*							
Complete genome	24.9	41.6	20.3	13.2	66.5	33.5	16,905
rrnS	33.6	31.5	22.0	13.0	65.1	35.0	911
rrnL	37.0	32.3	18.6	12.1	69.3	30.7	1728
Control region position: 10,853–11,762	23.3	35.4	21.8	19.6	58.7	41.4	910
Control region position: 16,105–16,905	25.7	34.5	23.5	16.4	60.2	39.9	801
*Oculina patagonica*							
Complete genome	25.3	42.9	19.6	12.3	68.2	31.9	14,856
rrnS	34.2	31.5	22.0	12.3	65.7	34.3	909
rrnL	37.3	32.2	18.5	11.9	69.5	30.4	1731
Control region position: 1910–2763	37.6	32.1	16.5	13.7	69.7	30.2	109
*Stephanocoenia intersepta*							
Complete genome	25.5	37.6	23.6	13.3	63.1	36.9	19,461
rrnS	31.6	28.6	24.7	15.1	60.2	39.8	961
rrnL_0‐a	34.0	29.3	24.2	12.4	63.3	36.6	491
rrnL_0‐b	31.5	30.7	22.8	14.9	62.2	37.7	1215
Control region position: 5761–6702	28.0	36.3	25.3	10.4	64.3	35.7	942
Control region position: 7385–8065	28.9	33.0	25.4	12.6	61.9	38	679

**FIGURE 2:**
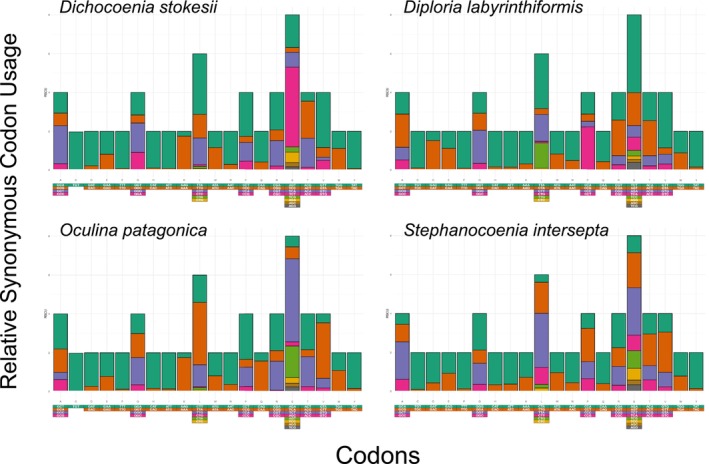
Relative synonymous codon usage (RSCU) profiles in the four studied mitochondrial genomes.

In two of the four studied species, *O. patagonica* and *Dicho. stokesii*, only ATG and ATA were used as start codons by PCGs, while in *Diplo. labyrinthiformis* and *S. intersepta* , start codons included ATG, ATA, and GTG. In all of the species studied, only two stop codons were observed: TAG and TAA (Tables S1–S4). Codon usage and relative synonymous codon usage (RSCU) were similar among the four studied coral species. The most frequently used codon in all of the species was TTT, that translated into Phenylalanine (used 464, 472, 496, and 310 times in *O. patagonica* , *Diplo. labyrinthiformis*, *Dicho. stokesii*, and *S. intersepta* , respectively). The second most frequent codon was TTA (used 318, 286, 313, and 271 times, respectively) followed by GTT (used 257, 259, 254, and 193 times, respectively) in all species studied (Tables S5–S8). In turn, other than stop codons, the least frequently used codon in all of the species was TGC (used 0, 3, 1, and 2 times in *O. patagonica* , *Diplo. labyrinthiformis*, *Dicho. stokesii*, and *S. intersepta* , respectively), followed by CGC (used 1, 4, 3, and 4 times, respectively) in the four species studied (Tables S5–S8). The RSCU analysis indicated, with only a few exceptions, a preference for A, T, or A + T rich codons among synonymous codons (Figure 2).

**FIGURE 3:**
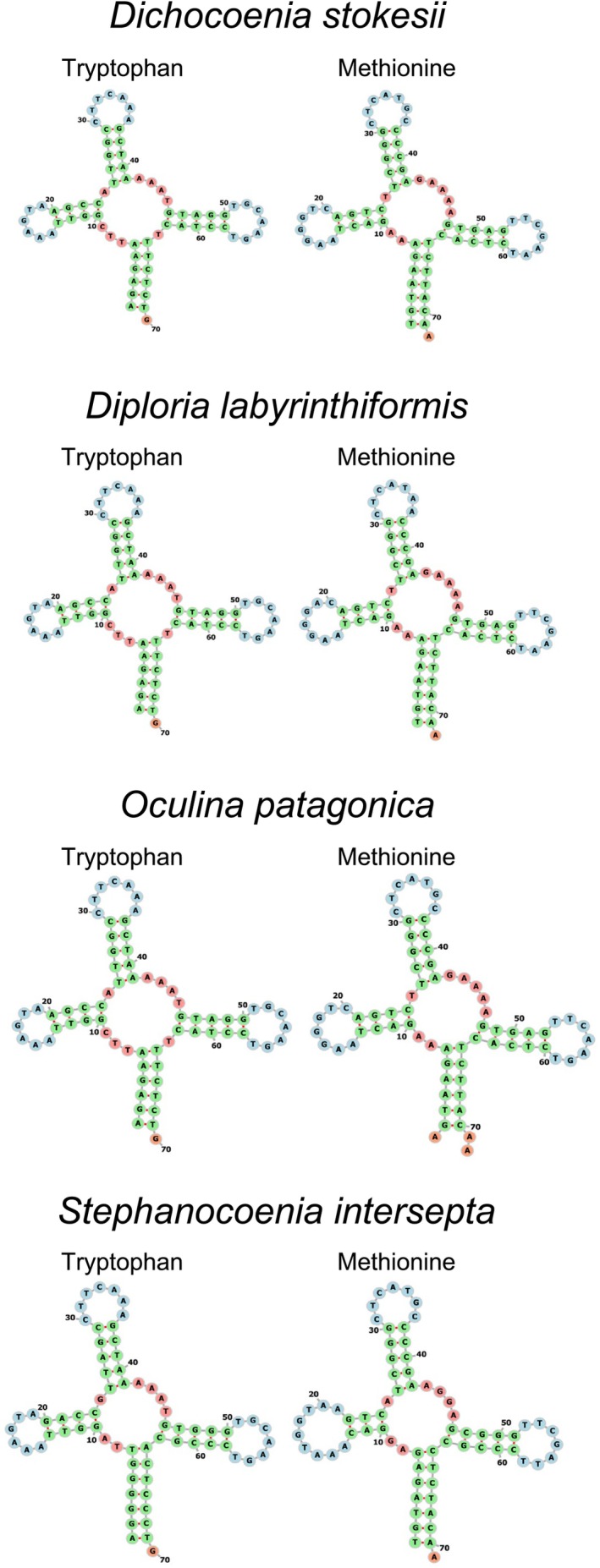
Clover‐leaf secondary structures of tRNAs in the four studied mitochondrial genomes annotated by Mitos2 on GalaxyEU website (https://usegalaxy.eu/) and predicted by TBI Forna website (http://rna.tbi.univie.ac.at/forna/).

The four studied mitochondrial genomes each contained two tRNAs (trnW—Tryptophan and trnM—Methionine), and these two tRNA genes exhibited the classic cloverleaf secondary structure (Figure 3). In the four studied genomes, trnW was 70 bp in length while trnM was 72 bp long in *Dicho. stokesii*, *Diplo. labyrinthiformis*, and *O. patagonica* and 71 bp long in *S. intersepta* . In line with our observations, the great majority of corals in which tRNAs have been analyzed have been shown to encode trnM and trnW, each with a cloverleaf secondary structure (Chen, Dai, et al. 2008; Flot and Tillier 2007; Niu et al. 2018; Tucker et al. 2023; Xiao et al. 2021; van Oppen et al. 2002). However, there are also coral species with contain 3 tRNA genes; trnM and two copies of trnW (e.g., *Seriatophora caliendrum* and *Stylophora pistillata* —Chen, Chiou, et al. 2008; *Seriatopora hystrix* —Chen, Dai, et al. 2008).

Three of the four studied mitochondrial genomes, *Dicho. stokesii*, *Diplo. labyrinthiformis*, and *O. patagonica* , contained one rrnS (12S) and one rrnL (16S) gene with an AT nucleotide skew. The A + T content estimated for the rrnS gene in *Dicho. stokesii*, *Diplo. labyrinthiformis*, and *O. patagonica* were 66.3%, 65.1%, and 69.3% respectively (Table 1). In turn, the A + T content estimated for the rrnL gene in *Dicho. stokesii*, *Diplo. labyrinthiformis*, and *O. patagonica* were 69.9%, 69.3%, and 69.5% respectively (Table 1). Interestingly, *S.intersepta* contained one rrnS (12S) and a bisected rrnL (16S) gene (rrnL_0‐a, rrnL_0‐b) with an AT content equal to 60.2%, 63.3%, and 62.2% respectively (Table 1). These newly assembled ribosomal genes serve as a reference to conduct biomonitoring of these and other imperiled corals in the greater Caribbean basin using eDNA.

The mitochondrial genomes of *Dicho. stokesii* and *O. patagonica* each contain a single long non‐coding 1843 bp (located between *cox1* and trnM) and 108 bp in length (located between *nad5* (5′) and *nad1*), respectively. In turn, the mitochondrial genomes of *Diplo. labyrinthiformis* and *S. intersepta* each contain two long non‐coding regions. In *Diplo. labyrinthiformis*, a 910 bp long non‐coding region was located between *cox3* and *cox2*, and a second 801 bp long non‐coding region was located between *cox1* and trnM. In *S. intersepta* , a 942 bp long non‐coding region was located between *cox1*‐a and *cox*‐1b while a second 679 bp long non‐coding region was located between *atp6* and *nad4*. In all four studied species, non‐coding regions were A+T‐rich (Table 1). The non‐coding regions for *Dicho. stokesii*, *Diplo. labyrinthiformis*, *O. patagonica* , and the shortest non‐coding region of *S. intersepta* had lower A+T contents than their respective entire mitochondrial genomes. In turn, the shortest non‐coding region of *S. intersepta* had a greater A+T content than their respective entire mitochondrial genomes (Table 1). A+T content is variable in the non‐coding regions of corals. For instance, non‐coding regions in *Montipora aequituberculata* , *Madracis myriaster* , and *Montipora vietnamensis* have a slightly lower A+T content than the respective entire mitochondrial genomes (Ju et al. 2017; Tucker et al. 2023; Wang et al. 2022). In turn, in *Pseudosiderastrea tayami* and *Pseudosiderastrea formosa*, a slightly greater A+T content is observed in its non‐coding regions compared to that of the entire mitochondrial genome (Chuang and Chen 2016).

The non‐coding regions of *Dicho. stokesii*, and *O. patagonica* contain 23 and 2 microsatellites, respectively, and most or all of them (21 and 2, respectively) were A+T rich. The longest control region of *Diplo. labyrinthiformis* contained 9 microsatellites, 7 of which were A + T rich while the shortest control region contained 2 microsatellites, 2 of which were A+T rich. The longer control region of *S. intersepta* contained 11 microsatellites, 9 of which were A + T rich; the smaller control region contained 6 microsatellites, 3 of which were AT rich (Table S9).

Short tandem repeats were also detected in most non‐coding regions of the studied mitochondrial genomes. The single non‐coding region of *Dicho. stokesii* exhibited two imperfect tandem repeats; one 50 bp long repeated 3.2 times and a second 52 bp long repeated 2.3 times (Table S10). There were no tandem repeats found in the non‐coding region of *O. patagonica* . The putative control regions in *Diplo. labyrinthiformis* have one tandem repeat each; the longest non‐coding region, located between *cox2* and *cox3*, featured one tandem repeat 15 bp repeated 3.7 times while the shortest non‐coding region exhibited a 27 bp in length repeated 2.1 times. Lastly, in *Stephanocoenia intersepta* , only a single non‐coding region had tandem repeats; the longest one located between *cox1*‐a and *cox1*‐b had an imperfect tandem repeat 23 bp long repeated 2.3 times (Table S10). All tandem repeats detected in *Dicho. stokesii*, *Diplo. labyrinthiformis*, *O. patagonica* , and *S. intersepta* were T‐rich.

The secondary structures of all non‐coding regions in *Dicho. stokesii*, *Diplo. labyrinthiformis*, *O. patagonica* , and *S. intersepta* contained hairpin structures throughout (Figure S1). A + T rich repetitive elements such as microsatellites and tandem repeats, and numerous hairpin structures in the secondary structure, have been observed in the non‐coding regions of the corals *Madracis myriaster* , *Acropora tenuis* , and *Montipora vietnamensis* in line with our observations (Tucker et al. 2023; van Oppen et al. 2002; Wang et al. 2022).

Overall, non‐coding regions in corals have rarely been examined in detail in scleractinian corals, with only 8 previously published papers describing them in detail (Chen, Chiou, et al. 2008; Chuang and Chen 2016; Flot and Tillier 2007; Ju et al. 2017; Tseng et al. 2005; Tucker et al. 2023; van Oppen et al. 2002; Wang et al. 2022). The well‐studied mitochondrial non‐coding regions vary in length and location, but all contain repeated motifs. Each non‐coding region is a candidate for the region of the mitochondrial genome in charge of DNA replication initiation (Flot and Tillier 2007). The shortest documented coral non‐coding regions are 627 bp in length (in *Montipora vietnamensis*—Wang et al. 2022; *Montipora cactus* —Tseng et al. 2005; *Anacropora matthai* —Tseng et al. 2005), and the longest coral non‐coding region is 1086 bp reported for *Acropora tenuis* (van Oppen et al. 2002). Our identified non‐coding control region for *O. patagonica* (108 bp long) is significantly shorter than previously documented mitochondrial non‐coding regions. However, we note that among eumetazoans, the smallest identified non‐coding control region is 121 bp in the sea urchin *Strongylocentrotus purpuratus* (Jacobs et al. 1988). Previously documented locations of non‐coding control regions for corals include between *nad4* and *atp8* ( *Madracis myriaster* —Tucker et al. 2023), between rrnS and *cox3* (*Montipora vietnamensis*—Wang et al. 2022; *Montipora aequituberculata* —Ju et al. 2017; *Montipora cactus* , *Anacropora matthai* —Tseng et al. 2005; *Acropora tenuis* —van Oppen et al. 2002), between *cob* and *nad2* (*Pseudosiderastrea tatami* and *Pseudosiderastrea formosa*—Chuang and Chen 2016), between *atp6* and *nad4* ( *Madracis mirabilis* —Chen, Chiou, et al. 2008), and between *atp8* and *cox1* (*Pocillopora grandis*—Flot and Tillier 2007). Comparative and functional studies are needed to improve our understanding of CR organization and function in coral mitochondrial genomes.

### 3.1 Phylomitogenomics of Scleractinian Corals

**FIGURE 4:**
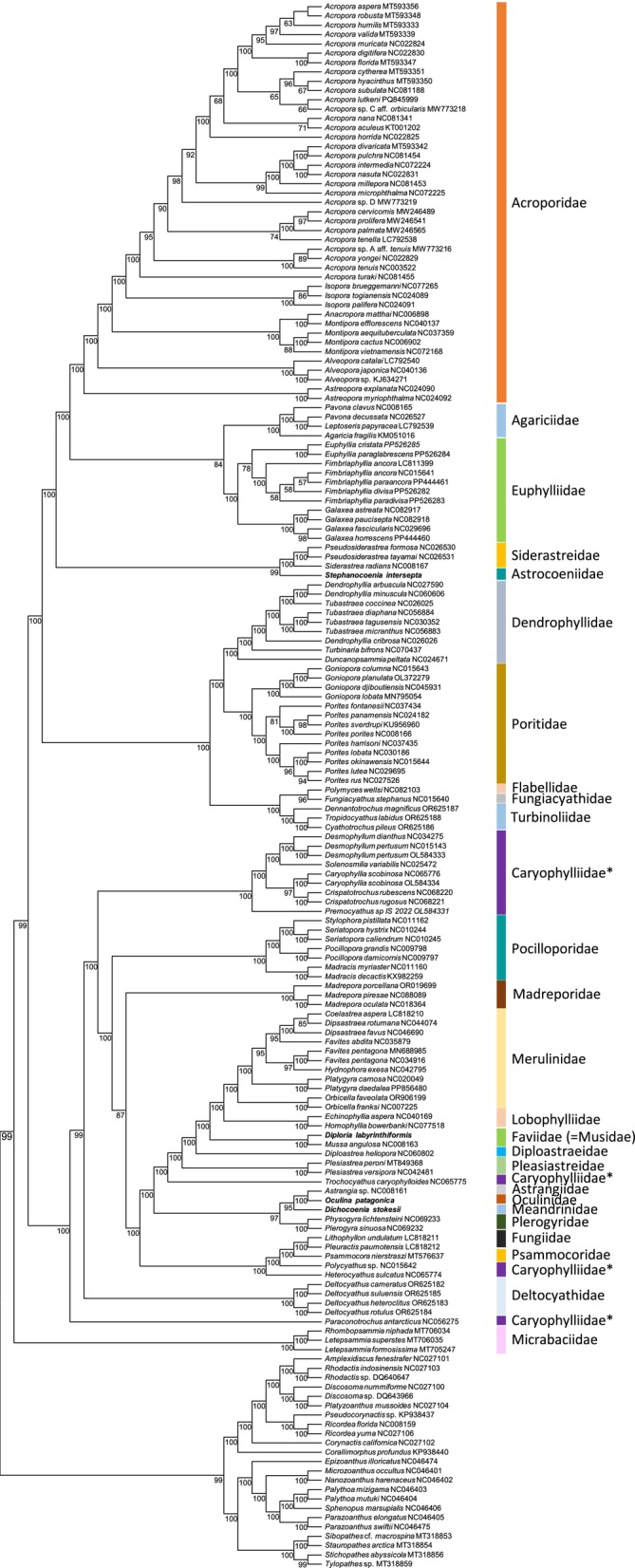
Phylogenetic tree (Maximum Likelihood) based on a concatenated alignment of amino acids of the 13 mitochondrial protein‐coding genes belonging to *Dichocoenia stokesii*, *Diploria labyrinthiformis* , *Oculina patagonica* , and *Stephanocoenia intersepta* plus 164 other species in the Order Scleractinia. Outgroups included 23 species belonging to the Class Hexacoralia but not belonging to the Order Scleractinia. The robustness of the ML tree was ascertained using 1000 bootstrap replicates (numbers below the nodes) of the tree search. Asterisks indicate the different species belonging to the family Caryophylliidae that were not resolved as a monophyletic clade in the analysis.

The phylogenetic analysis (ML, 11,502 nucleotide sites, of which 6930 were parsimony informative) supported the monophyletic status of the Order Scleractinia, taking into account that the four newly assembled mitochondrial genomes belonging to *Dicho. stokesii*, *Diplo. labyrinthiformis*, *O. patagonica* , and *S. intersepta* , plus those of all other species of stony corals (Scleractinia) used in the phylogenetic analysis, clustered together into a single well‐supported (bootstrap value [bv] = 99) clade (Figure 4). Within the monophyletic Order Scleractinia, and when taking only into consideration families represented by at least two genera in our analysis, the families Acroporidae, Agariciidae, Deltocyathidae, Dendrophyllidae, Euphylliidae, Faviidae (=Musidae), Fungidae, Lobophylliidae, Merulinidae, Micrabaciidae, Plerogyridae, Pocilloporidae, Poritidae, Siderastreidae, and Turbinoliidae were fully supported (bv = 100) as monophyletic in our ML phylogenetic tree. In turn, the family Caryophylliidae was polyphyletic (Figure 4).

In the ML tree, *S. intersepta* , belonging to the family Astrocoeniidae, was well supported (bv = 99) as a clade sister to the family Siderastreidae while *Diplo. labyrinthiformis* clustered together with *Mussa angulosa* and supported the status of the family Faviidae (=Musidae, bv = 100). *Oculina patagonica* (fam. Oculinidae) was sister to *Astrangia* sp. (fam. Astrangiidae) (bv = 100) while *Dicho. stokesii* (fam. Meandrinidae) was sister to the fully supported clade containing *O. patagonica* and *Astrangia* sp.

Overall, our ML analysis supported most relationships among families of hard corals reported by the most recent phylogenetic and phylogenomic studies (Seiblitz et al. 2020, 2022; Tian et al. 2021, 2022; Xiao et al. 2021; Vaga et al. 2022; Tucker et al. 2023). We argue in favor of additional studies sequencing mitochondrial genomes of scleractinian corals to explore phylogenetic relationships in‐depth in this clade. Research to sequence and analyze in detail the mitochondrial genome of scleractinian corals inhabiting the greater Caribbean basin is underway and such studies will also support biomonitoring of these imperiled sessile invertebrates using eDNA.

## Author Contributions

**Katrina Zabransky:** data curation (equal), formal analysis (equal), investigation (equal), methodology (equal), visualization (equal), writing – original draft (equal), writing – review and editing (equal). **William Vuong:** formal analysis (equal), investigation (equal), methodology (equal), visualization (equal), writing – original draft (equal), writing – review and editing (equal). **Stephanie M. Rosales:** conceptualization (equal), data curation (equal), formal analysis (equal), funding acquisition (equal), investigation (equal), project administration (equal), resources (equal), writing – review and editing (equal). **J. Antonio Baeza:** conceptualization (equal), data curation (equal), formal analysis (equal), funding acquisition (equal), investigation (equal), methodology (equal), project administration (equal), resources (equal), supervision (equal), validation (equal), visualization (equal), writing – original draft (equal), writing – review and editing (equal).

## Conflicts of Interest

The authors declare no conflicts of interest.

## Supporting information


**Data S1:** ece372967‐sup‐0001‐Supinfo.docx.

## Data Availability

Illumina sequencing reads are available in the NCBI Sequence Read Archive (SRA) repository with SRA accessions SRR15960007 ( *Diploria labyrinthiformis* ), SRR15960011 (*Dichocoenia stokesii*), SRR15960036 ( *Stephanocoenia intersepta* ), and SRR28110921 ( *Oculina patagonica* ). The assembled mitochondrial genomes have been deposited and are available in the NCBI nucleotide repository with accession numbers PV487494 (*Dichocoenia stokesii*), PV487495 ( *Diploria labyrinthiformis* ), PV487496 ( *Oculina patagonica* ), and PV423450 ( *Stephanocoenia intersepta* ). Samples were collected under permits #FKNMS‐2018‐057 and #FKNMS‐2017‐100 to S.R.
