# Supplementary material for: Genomic Resources for Imperiled Caribbean Reef‐Forming Corals (Hexacorallia: Scleractinia): Complete Mitochondrial Genomes of Dichocoenia stokesii, Diploria labyrinthiformis, Oculina patagonica, and Stephanocoenia intersepta
Source: Ecol Evol. 2026 Jan 30;16(2):e72967. doi: 10.1002/ece3.72967 (PMC12856378; doi:10.1002/ece3.72967)
Supplement: Supplementary file 1 — Data S1: ece372967‐sup‐0001‐Supinfo.docx. [file ECE3-16-e72967-s001.docx]

**Genomic Resources for imperiled Caribbean reef-forming corals (Hexacorallia: Scleractinia): Complete mitochondrial genomes of *Dichocoenia stokesii, Diploria labyrinthiformis, Oculina patagonica,* and *Stephanocoenia intersepta***

Katrina Zabransky^a^, William Vuong^a^, Stephanie M. Rosales^b,c^, J. Antonio Baeza^a,c,d^

^a^ Department of Biological Sciences, Clemson University, Clemson, SC, USA

^b^ The University of Miami, Cooperative Institute for Marine and Atmospheric Studies, Miami, FL, USA

^c^ National Oceanic and Atmospheric Administration, Atlantic Oceanographic and Meteorological Laboratory, Miami, FL, USA

^d^ Smithsonian Marine Station at Fort Pierce, Smithsonian Institution, Fort Pierce, FL, USA

^e^ Departamento de Biología Marina, Universidad Catolica del Norte, Coquimbo, Chile

*Corresponding author

Email: [baeza.antonio@gmail.com](mailto:baeza.antonio@gmail.com) (J.A. Baeza). Department of Biological Sciences, Clemson University, Clemson, SC, USA

**Supplementary Materials**

**Supplementary Table 1.** Mitochondrial genome of *Dichocoencia stokesii*: arrangement and annotation (17,171 bp)

| Name | Type | Start | Stop | Strand | Length (bp) | Start | Stop | Anticodon | Continuity |
| --- | --- | --- | --- | --- | --- | --- | --- | --- | --- |
| trnM (cat) | tRNA | 1 | 72 | + | 72 |  |  | CAT | +397 |
| rrnL | rRNA | 470 | 2192 | + | 1723 |  |  |  | +3 |
| OH_2-b |  | 2196 | 2257 | + | 62 |  |  |  | -24 |
| nad5 (5’) | PCG | 2234 | 2944 | + | 711 | ATG |  |  | -153 |
| gpI |  | 2934 | 3086 | + | 153 |  |  |  | -34 |
| nad1 | PCG | 3053 | 4000 | + | 948 | ATG | TAG |  | +2 |
| cob | PCG | 4003 | 5142 | + | 1140 | ATA | TAA |  | +70 |
| OH_0 |  | 5213 | 5257 | + | 45 |  |  |  | +108 |
| OH_2-c |  | 5366 | 5403 | + | 38 |  |  |  | -236 |
| nad2 | PCG | 5168 | 6454 | + | 1287 | ATA | TAA |  | 0 |
| nad6 | PCG | 6455 | 7015 | + | 561 | ATG | TAA |  | -1 |
| atp6 | PCG | 7015 | 7692 | + | 678 | ATG | TAA |  | -1 |
| nad4 | PCG | 7692 | 9131 | + | 1440 | ATG | TAG |  | -3 |
| rrnS | rRNA | 9129 | 10038 | + | 910 |  |  |  | -7 |
| cox3 | PCG | 10032 | 10811 | + | 780 | ATG | TAA |  | +169 |
| cox2 | PCG | 10981 | 11688 | + | 708 | ATG | TAG |  | -19 |
| nad4l | PCG | 11670 | 11969 | + | 300 | ATG | TAA |  | -1 |
| nad3 | PCG | 11969 | 12313 | + | 345 | ATG | TAA |  | +56 |
| nad5 (3’) | PCG | 12370 | 13473 | + | 1104 |  | TAG |  | -2 |
| trnW (tca) | tRNA | 13472 | 13541 | + | 70 |  |  | TCA | +3 |
| atp8 | PCG | 13545 | 13742 | + | 198 | ATG | TAA |  | -1 |
| cox1 | PCG | 13742 | 15328 | + | 1587 | ATG | TAA |  | -29 |
| OH_1 |  | 15300 | 15343 | + | 44 |  |  |  | +1004 |
| OH_2-a |  | 16348 | 16386 | + | 39 |  |  |  | +786 |

**Supplementary Table 2.** Mitochondrial genome of *Diploria labyrinthiformis*: arrangement and annotation (16,905 bp)

| Name | Type | Start | Stop | Strand | Length (bp) | Start | Stop | Anticodon | Continuity |
| --- | --- | --- | --- | --- | --- | --- | --- | --- | --- |
| trnM (cat) | tRNA | 1 | 72 | + | 72 |  |  | CAT | +110 |
| rrnL | rRNA | 183 | 1910 | + | 1728 |  |  |  | -1267 |
| OH_2-a |  | 644 | 707 | + |  |  |  |  | +257 |
| OH_3-b |  | 965 | 1013 | + |  |  |  |  | +578 |
| OH_1-b |  | 1592 | 1801 | + |  |  |  |  | +196 |
| nad5 (5’) | PCG | 1998 | 2708 | + | 711 | ATG |  |  | -92 |
| OH_2-b |  | 2617 | 2645 | + |  |  |  |  | +52 |
| gpI |  | 2698 | 2857 | + |  |  |  |  | -40 |
| nad1 | PCG | 2818 | 3765 | + | 948 | ATG | TAG |  | +2 |
| cob | PCG | 3768 | 4907 | + | 1140 | ATG | TAA |  | +69 |
| OH_1-a |  | 4977 | 5022 | + |  |  |  |  | -90 |
| nad2 | PCG | 4933 | 6219 | + | 1287 | ATA | TAA |  | +1 |
| nad6 | PCG | 6221 | 6781 | + | 561 | ATA | TAA |  | -1 |
| atp6 | PCG | 6781 | 7458 | + | 678 | ATG | TAA |  | -1 |
| nad4 | PCG | 7458 | 8897 | + | 1440 | ATG | TAG |  | +136 |
| rrnS | rRNA | 9034 | 9944 | + | 911 |  |  |  | -7 |
| OH_0 |  | 9938 | 10073 | + |  |  |  |  | -1 |
| cox3 | PCG | 10073 | 10852 | + | 780 | GTG | TAA |  | -34 |
| OH_3-a |  | 10819 | 10866 | + |  |  |  |  | +896 |
| cox2 | PCG | 11763 | 12470 | + | 708 | ATG | TAG |  | -19 |
| nad4l | PCG | 12452 | 12751 | + | 300 | ATG | TAA |  | +2 |
| nad3 | PCG | 12754 | 13095 | + | 342 | ATG | TAA |  | +44 |
| nad5 (3’) | PCG | 13140 | 14255 | + | 1116 |  | TAG |  | -2 |
| trnW (tca) | tRNA | 14254 | 14323 | + | 70 |  |  | TCA | +3 |
| atp8 | PCG | 14327 | 14524 | + | 198 | ATG | TAA |  | -1 |
| cox1 | PCG | 14524 | 16104 | + | 1581 | ATG | TAA |  | +801 |

**Supplementary Table 3.** Mitochondrial genome of *Stephanocoenia intersepta*: arrangement and annotation (19,461 bp)

| Name | Type | Start | Stop | Strand | Length (bp) | Start  Codn | Stop  Codn | Anticodon | Continuity |
| --- | --- | --- | --- | --- | --- | --- | --- | --- | --- |
| trnM (cat) | tRNA | 1 | 71 | + | 71 |  |  | CAT | +246 |
| rrnL_0-a | rRNA | 318 | 808 | + | 491 |  |  |  | +34 |
| cox3 | PCG | 843 | 1631 | + | 789 | ATG | TAG |  | +36 |
| cox2 | PCG | 1668 | 2405 | + | 738 | ATG | TAG |  | +55 |
| nad4l | PCG | 2459 | 2758 | + | 300 | GTG | TAA |  | +18 |
| nad3 | PCG | 2775 | 3131 | + | 357 | GTG | TAG |  | -35 |
| gpI |  | 3097 | 3232 | + | 136 |  |  |  | +20 |
| nad5 (3’) | PCG | 3253 | 4371 | + | 1119 |  | TAA |  | +41 |
| trnW (tca) | tRNA | 4413 | 4482 | + | 70 |  |  | TCA | +90 |
| OH-a |  | 4573 | 4603 | + | 31 |  |  |  | -75 |
| atp8 | PCG | 4529 | 4759 | + | 231 | ATG | TAA |  | +107 |
| cox1-a | PCG | 4867 | 5760 | + | 894 | ATG |  |  | -10 |
| gpI |  | 5751 | 6012 | + | 262 |  |  |  | +690 |
| cox1-b | PCG | 6703 | 7386 | + | 684 |  | TAA |  | +679 |
| rrnL_0-b | rRNA | 8066 | 9280 | + | 1215 |  |  |  | +384 |
| nad5 (5’) | PCG | 9665 | 10387 | + | 723 | GTG |  |  | +415 |
| nad1 | PCG | 10803 | 11747 | + | 945 | ATG | TAA |  | +134 |
| cob | PCG | 11882 | 13036 | + | 1155 | ATG | TAG |  | +309 |
| nad2 | PCG | 13346 | 14566 | + | 1221 | ATA | TAA |  | +65 |
| nad6 | PCG | 14632 | 15192 | + | 561 | ATA | TAA |  | +45 |
| atp6 | PCG | 15238 | 15936 | + | 699 | ATG | TAG |  | +16 |
| OH-b |  | 15953 | 15993 | + | 41 |  |  |  | +446 |
| nad4 | PCG | 16440 | 17867 | + | 1428 | ATG | TAA |  | +242 |
| rrnS | rRNA | 18110 | 19070 | + | 961 |  |  |  | +392 |

**Supplementary Table 4.** Mitochondrial genome of *Oculina patagonica*: arrangement and annotation (14,856 bp)

| Name | Type | Start | Stop | Strand | Length (bp) | Start | Stop | Anticodon | Continuity |
| --- | --- | --- | --- | --- | --- | --- | --- | --- | --- |
| trnM (cat) | tRNA | 1 | 72 | + | 72 |  |  | CAT | +106 |
| rrnL | rRNA | 179 | 1909 | + | 1731 |  |  |  | -1274 |
| OH_0-c |  | 636 | 684 | + | 49 |  |  |  | +162 |
| OH_0-1b |  | 847 | 885 | + | 39 |  |  |  | +704 |
| OH_0-e |  | 1590 | 1799 | + | 210 |  |  |  | +107 |
| OH_0-a |  | 1907 | 1968 | + | 62 |  |  |  | -24 |
| nad5 (5’) | PCG | 1945 | 2655 | + | 711 | GTG |  |  | -92 |
| OH_0-d |  | 2564 | 2592 | + | 29 |  |  |  | +52 |
| gpI |  | 2645 | 2797 | + | 153 |  |  |  | -34 |
| nad1 | PCG | 2764 | 3711 | + | 948 | ATG | TAG |  | +2 |
| cob | PCG | 3714 | 4853 | + | 1140 | ATG | TAG |  | +70 |
| OH_0-b |  | 4924 | 4968 | + | 45 |  |  |  | -90 |
| nad2 | PCG | 4879 | 6165 | + | 1287 | ATA | TAA |  | -9 |
| OH_1-a |  | 6157 | 6195 | + | 39 |  |  |  | -30 |
| nad6 | PCG | 6166 | 6726 | + | 561 | ATG | TAA |  | -1 |
| atp6 | PCG | 6726 | 7403 | + | 678 | ATG | TAA |  | -1 |
| nad4 | PCG | 7403 | 8842 | + | 1440 | ATG | TAG |  | -4 |
| rrnS | rRNA | 8839 | 9747 | + | 909 |  |  |  | -289 |
| gpI |  | 9459 | 9598 | + | 140 |  |  |  | +143 |
| cox3 | PCG | 9742 | 10521 | + | 780 | ATG | TAA |  | +2 |
| cox2 | PCG | 10524 | 11231 | + | 708 | ATG | TAG |  | -19 |
| nad4l | PCG | 11213 | 11512 | + | 300 | ATG | TAA |  | -1 |
| nad3 | PCG | 11512 | 11856 | + | 345 | ATG | TAA |  | +57 |
| nad5 (3’) | PCG | 11914 | 13017 | + | 1104 |  | TAG |  | -2 |
| trnW (tca) | tRNA | 13016 | 13085 | + | 70 |  |  | TCA | +3 |
| atp8 | PCG | 13089 | 13286 | + | 198 | ATG | TAA |  | -1 |
| cox1 | PCG | 13286 | 14836 | + | 1551 | ATG | TAA |  | +21 |

**Supplementary Table S5.** Codon usage in protein-coding genes of *Dichocoenia stokesii*. Stop codons were not included in the analysis. Results for 11,190 residue sequences

AmAcid Codon Number /1000 Fraction ..

Ala GCG 35.00 9.38 0.16

Ala GCA 58.00 15.55 0.27

Ala GCT 107.00 28.69 0.49

Ala GCC 18.00 4.83 0.08

Cys TGT 37.00 9.92 1.00

Cys TGC 0.00 0.00 0.00

Asp GAT 72.00 19.30 0.89

Asp GAC 9.00 2.41 0.11

Glu GAG 43.00 11.53 0.41

Glu GAA 62.00 16.62 0.59

Phe TTT 466.00 124.93 0.96

Phe TTC 17.00 4.56 0.04

Gly GGG 113.00 30.29 0.38

Gly GGA 69.00 18.50 0.23

Gly GGT 87.00 23.32 0.29

Gly GGC 31.00 8.31 0.10

His CAT 69.00 18.50 0.95

His CAC 4.00 1.07 0.05

Ile ATT 225.00 60.32 0.96

Ile ATC 9.00 2.41 0.04

Lys AAG 14.00 3.75 0.12

Lys AAA 100.00 26.81 0.88

Leu TTG 114.00 30.56 0.20

Leu TTA 293.00 78.55 0.52

Leu CTG 6.00 1.61 0.01

Leu CTA 12.00 3.22 0.02

Leu CTT 129.00 34.58 0.23

Leu CTC 8.00 2.14 0.01

Met ATG 98.00 26.27 0.58

Met ATA 71.00 19.03 0.42

Asn AAT 81.00 21.72 0.85

Asn AAC 14.00 3.75 0.15

Pro CCG 16.00 4.29 0.12

Pro CCA 33.00 8.85 0.24

Pro CCT 78.00 20.91 0.57

Pro CCC 11.00 2.95 0.08

Gln CAG 14.00 3.75 0.21

Gln CAA 54.00 14.48 0.79

Arg CGG 8.00 2.14 0.14

Arg CGA 28.00 7.51 0.48

Arg CGT 19.00 5.09 0.33

Arg CGC 3.00 0.80 0.05

Ser AGG 6.00 1.61 0.02

Ser AGA 20.00 5.36 0.07

Ser AGT 62.00 16.62 0.21

Ser AGC 8.00 2.14 0.03

Ser TCG 10.00 2.68 0.03

Ser TCA 28.00 7.51 0.10

Ser TCT 151.00 40.48 0.51

Ser TCC 9.00 2.41 0.03

Thr ACG 15.00 4.02 0.11

Thr ACA 50.00 13.40 0.37

Thr ACT 64.00 17.16 0.48

Thr ACC 5.00 1.34 0.04

Val GTG 42.00 11.26 0.12

Val GTA 42.00 11.26 0.12

Val GTT 239.00 64.08 0.71

Val GTC 14.00 3.75 0.04

Trp TGG 40.00 10.72 0.44

Trp TGA 50.00 13.40 0.56

Tyr TAT 135.00 36.19 0.96

Tyr TAC 5.00 1.34 0.04

End TAG 0.00 0.00 0.00

End TAA 0.00 0.00 0.00

**Supplementary Table 6.** Codon usage in protein-coding genes of *Diploria labyrinthiformis*. Stop codons were not included in the analysis. Results for 11,739 residue sequences

AmAcid Codon Number /1000 Fraction ..

Ala GCG 37.00 9.46 0.16

Ala GCA 63.00 16.10 0.28

Ala GCT 97.00 24.79 0.43

Ala GCC 30.00 7.67 0.13

Cys TGT 49.00 12.52 0.94

Cys TGC 3.00 0.77 0.06

Asp GAT 60.00 15.33 0.76

Asp GAC 19.00 4.86 0.24

Glu GAG 49.00 12.52 0.44

Glu GAA 63.00 16.10 0.56

Phe TTT 472.00 120.62 0.95

Phe TTC 25.00 6.39 0.05

Gly GGG 136.00 34.76 0.43

Gly GGA 70.00 17.89 0.22

Gly GGT 85.00 21.72 0.27

Gly GGC 28.00 7.16 0.09

His CAT 68.00 17.38 0.92

His CAC 6.00 1.53 0.08

Ile ATT 222.00 56.73 0.93

Ile ATC 18.00 4.60 0.07

Lys AAG 19.00 4.86 0.16

Lys AAA 99.00 25.30 0.84

Leu TTG 131.00 33.48 0.22

Leu TTA 286.00 73.09 0.47

Leu CTG 9.00 2.30 0.01

Leu CTA 30.00 7.67 0.05

Leu CTT 139.00 35.52 0.23

Leu CTC 10.00 2.56 0.02

Met ATG 100.00 25.56 0.58

Met ATA 71.00 18.14 0.42

Asn AAT 79.00 20.19 0.75

Asn AAC 26.00 6.64 0.25

Pro CCG 13.00 3.32 0.09

Pro CCA 39.00 9.97 0.28

Pro CCT 78.00 19.93 0.56

Pro CCC 10.00 2.56 0.07

Gln CAG 15.00 3.83 0.22

Gln CAA 54.00 13.80 0.78

Arg CGG 7.00 1.79 0.12

Arg CGA 27.00 6.90 0.46

Arg CGT 21.00 5.37 0.36

Arg CGC 4.00 1.02 0.07

Ser AGG 7.00 1.79 0.02

Ser AGA 23.00 5.88 0.07

Ser AGT 65.00 16.61 0.21

Ser AGC 6.00 1.53 0.02

Ser TCG 15.00 3.83 0.05

Ser TCA 26.00 6.64 0.08

Ser TCT 154.00 39.36 0.50

Ser TCC 11.00 2.81 0.04

Thr ACG 19.00 4.86 0.13

Thr ACA 53.00 13.54 0.36

Thr ACT 66.00 16.87 0.45

Thr ACC 8.00 2.04 0.05

Val GTG 46.00 11.76 0.13

Val GTA 30.00 7.67 0.08

Val GTT 259.00 66.19 0.71

Val GTC 28.00 7.16 0.08

Trp TGG 49.00 12.52 0.56

Trp TGA 39.00 9.97 0.44

Tyr TAT 131.00 33.48 0.92

Tyr TAC 11.00 2.81 0.08

End TAG 0.00 0.00 0.00

End TAA 0.00 0.00 0.00

**Supplementary Table S7.** Codon usage in protein-coding genes of *Oculina patagonica*. Stop codons were not included in the analysis. Results for 11,712 residue sequences

AmAcid Codon Number /1000 Fraction ..

Ala GCG 36.00 9.22 0.15

Ala GCA 71.00 18.19 0.30

Ala GCT 106.00 27.15 0.45

Ala GCC 22.00 5.64 0.09

Cys TGT 42.00 10.76 1.00

Cys TGC 0.00 0.00 0.00

Asp GAT 72.00 18.44 0.87

Asp GAC 11.00 2.82 0.13

Glu GAG 42.00 10.76 0.39

Glu GAA 66.00 16.91 0.61

Phe TTT 464.00 118.85 0.94

Phe TTC 30.00 7.68 0.06

Gly GGG 112.00 28.69 0.35

Gly GGA 81.00 20.75 0.25

Gly GGT 99.00 25.36 0.31

Gly GGC 28.00 7.17 0.09

His CAT 67.00 17.16 0.92

His CAC 6.00 1.54 0.08

Ile ATT 229.00 58.66 0.93

Ile ATC 17.00 4.35 0.07

Lys AAG 15.00 3.84 0.13

Lys AAA 103.00 26.38 0.87

Leu TTG 112.00 28.69 0.19

Leu TTA 318.00 81.45 0.53

Leu CTG 5.00 1.28 0.01

Leu CTA 13.00 3.33 0.02

Leu CTT 140.00 35.86 0.24

Leu CTC 7.00 1.79 0.01

Met ATG 105.00 26.90 0.60

Met ATA 71.00 18.19 0.40

Asn AAT 85.00 21.77 0.82

Asn AAC 19.00 4.87 0.18

Pro CCG 14.00 3.59 0.10

Pro CCA 35.00 8.97 0.25

Pro CCT 82.00 21.00 0.59

Pro CCC 9.00 2.31 0.06

Gln CAG 15.00 3.84 0.21

Gln CAA 55.00 14.09 0.79

Arg CGG 8.00 2.05 0.14

Arg CGA 28.00 7.17 0.47

Arg CGT 22.00 5.64 0.37

Arg CGC 1.00 0.26 0.02

Ser AGG 7.00 1.79 0.02

Ser AGA 21.00 5.38 0.07

Ser AGT 61.00 15.63 0.20

Ser AGC 8.00 2.05 0.03

Ser TCG 9.00 2.31 0.03

Ser TCA 23.00 5.89 0.08

Ser TCT 161.00 41.24 0.53

Ser TCC 11.00 2.82 0.04

Thr ACG 13.00 3.33 0.09

Thr ACA 56.00 14.34 0.39

Thr ACT 67.00 17.16 0.46

Thr ACC 9.00 2.31 0.06

Val GTG 45.00 11.53 0.13

Val GTA 42.00 10.76 0.12

Val GTT 257.00 65.83 0.71

Val GTC 16.00 4.10 0.04

Trp TGG 48.00 12.30 0.54

Trp TGA 41.00 10.50 0.46

Tyr TAT 135.00 34.58 0.92

Tyr TAC 11.00 2.82 0.08

End TAG 0.00 0.00 0.00

End TAA 0.00 0.00 0.00

**Supplementary Table S8.** Codon usage in protein-coding genes of *Stephanocoenia intersepta*. Stop codons were not included in the analysis. Results for 11,805 residue sequences

AmAcid Codon Number /1000 Fraction ..

Ala GCG 63.00 16.01 0.22

Ala GCA 39.00 9.91 0.14

Ala GCT 135.00 34.31 0.48

Ala GCC 44.00 11.18 0.16

Cys TGT 37.00 9.40 0.95

Cys TGC 2.00 0.51 0.05

Asp GAT 67.00 17.03 0.78

Asp GAC 19.00 4.83 0.22

Glu GAG 64.00 16.26 0.53

Glu GAA 56.00 14.23 0.47

Phe TTT 310.00 78.78 0.93

Phe TTC 23.00 5.84 0.07

Gly GGG 150.00 38.12 0.47

Gly GGA 54.00 13.72 0.17

Gly GGT 87.00 22.11 0.27

Gly GGC 29.00 7.37 0.09

His CAT 68.00 17.28 0.83

His CAC 14.00 3.56 0.17

Ile ATT 192.00 48.79 0.81

Ile ATC 44.00 11.18 0.19

Lys AAG 39.00 9.91 0.36

Lys AAA 68.00 17.28 0.64

Leu TTG 155.00 39.39 0.27

Leu TTA 271.00 68.87 0.46

Leu CTG 18.00 4.57 0.03

Leu CTA 38.00 9.66 0.07

Leu CTT 85.00 21.60 0.15

Leu CTC 17.00 4.32 0.03

Met ATG 121.00 30.75 0.52

Met ATA 113.00 28.72 0.48

Asn AAT 73.00 18.55 0.78

Asn AAC 21.00 5.34 0.22

Pro CCG 25.00 6.35 0.16

Pro CCA 29.00 7.37 0.19

Pro CCT 65.00 16.52 0.43

Pro CCC 33.00 8.39 0.22

Gln CAG 15.00 3.81 0.20

Gln CAA 61.00 15.50 0.80

Arg CGG 12.00 3.05 0.24

Arg CGA 22.00 5.59 0.44

Arg CGT 12.00 3.05 0.24

Arg CGC 4.00 1.02 0.08

Ser AGG 14.00 3.56 0.04

Ser AGA 36.00 9.15 0.11

Ser AGT 74.00 18.81 0.22

Ser AGC 10.00 2.54 0.03

Ser TCG 37.00 9.40 0.11

Ser TCA 33.00 8.39 0.10

Ser TCT 100.00 25.41 0.30

Ser TCC 25.00 6.35 0.08

Thr ACG 32.00 8.13 0.18

Thr ACA 46.00 11.69 0.26

Thr ACT 71.00 18.04 0.41

Thr ACC 26.00 6.61 0.15

Val GTG 89.00 22.62 0.24

Val GTA 70.00 17.79 0.19

Val GTT 193.00 49.05 0.52

Val GTC 22.00 5.59 0.06

Trp TGG 60.00 15.25 0.61

Trp TGA 39.00 9.91 0.39

Tyr TAT 152.00 38.63 0.93

Tyr TAC 12.00 3.05 0.07

End TAG 0.00 0.00 0.00

End TAA 0.00 0.00 0.00

**Supplementary Materials Table S9.** Microsatellites found within the long non-coding control regions of the complete mitochondrial genome of *Dichocoenia stokesii, Diploria labyrinthiformis, Oculina patagonica,* and *Stephanocoenia intersepta* from the web server BioPHP Microsatellite Repeats Finder using the default parameters (<http://insilico.ehu.es/mini_tools/microsatellites/> - Bikandi et al. 2004).

| **CR Position** | **Length** | **Period Size** | **Entire Sequence** |
| --- | --- | --- | --- |
| ***Dichocoenia stokesii*** (Genome Position: 15,329-17,171) | | | |
| 1 | 2 | 6 | AAAAAAAAAAAA |
| 97 | 2 | 3 | TTTTTT |
| 173 | 2 | 3 | TTTTTT |
| 311 | 2 | 3 | TTTTTT |
| 431 | 2 | 3 | GAGAGA |
| 588 | 2 | 3 | TATATA |
| 609 | 2 | 3 | TTTTTT |
| 682 | 2 | 3 | TATATA |
| 760 | 2 | 3 | TTTTTT |
| 795 | 2 | 4 | TTTTTTTT |
| 821 | 2 | 3 | GGGGGG |
| 888 | 2 | 3 | TATATA |
| 904 | 2 | 3 | TTTTTT |
| 1019 | 2 | 3 | TTTTTT |
| 1029 | 2 | 3 | AAAAAA |
| 1039 | 2 | 6 | AAAAAAAAAAAA |
| 1071 | 2 | 3 | TTTTTT |
| 1125 | 2 | 3 | TTTTTT |
| 1157 | 2 | 3 | TTTTTT |
| 1502 | 2 | 4 | TTTTTTTT |
| 1542 | 2 | 3 | ATATAT |
| 1724 | 2 | 3 | AAAAAA |
| 1802 | 2 | 3 | TTTTTT |
| ***Diploria labyrinthiformis*** (Genome Position: 10,853-11,762) | | | |
| 2 | 2 | 3 | TTTTTT |
| 75 | 2 | 4 | TTTTTTTT |
| 151 | 2 | 3 | AAAAAA |
| 190 | 2 | 3 | TTTTTT |
| 355 | 2 | 3 | CCCCCCC |
| 376 | 3 | 3 | GGAGGAGGA |
| 673 | 3 | 3 | TTCTTCTTC |
| 706 | 3 | 3 | TTCTTCTTC |
| 867 | 2 | 5 | TTTTTTTTTT |
| ***Diploria labyrinthiformis*** (Genome Position: 16,105-16,905) | | | |
| 44 | 2 | 3 | TTTTTT |
| 54 | 2 | 4 | TATATATA |
| ***Oculina patagonica*** (Genome Position: 2,655-2,763) | | | |
| 18 | 2 | 3 | AAAAAA |
| 30 | 2 | 3 | AAAAAA |
| ***Stephanocoenia intersepta*** (Genome Position: 5,761-6,702) | | | |
| 192 | 2 | 4 | TTTTTTTT |
| 282 | 2 | 4 | TTTTTTTT |
| 333 | 2 | 4 | AAAAAAAA |
| 415 | 2 | 3 | TATATA |
| 602 | 3 | 3 | TTATTATTA |
| 632 | 2 | 3 | GGGGGG |
| 736 | 2 | 3 | AAAAAA |
| 826 | 2 | 5 | AAAAAAAAAA |
| 839 | 2 | 3 | TTTTTT |
| 904 | 2 | 3 | GGGGGG |
| 932 | 2 | 3 | ATATAT |
| ***Stephanocoenia intersepta*** (Genome Position: 7,385-8,065) | | | |
| 42 | 2 | 3 | CTCTCT |
| 103 | 2 | 3 | TTTTTT |
| 123 | 2 | 3 | TTTTTT |
| 136 | 2 | 4 | TTTTTTTT |
| 343 | 2 | 3 | GGGGGG |
| 415 | 2 | 3 | GGGGGG |

**Supplementary Materials Table S10.** Tandem repeats found within the long non-coding control regions of the complete mitochondrial genome of *Dichocoenia stokesii, Diploria labyrinthiformis, Oculina patagonica,* and *Stephanocoenia intersepta* using the advanced parameters (2,3,5) of the web server Tandem Repeat Finder (<https://tandem.bu.edu/trf/trf.basic.submit.html>).

| **CR Position** | **Size (bp) of Motif** | **Copy Number** | **Consensus Sequence** |
| --- | --- | --- | --- |
| ***Dichocoenia stokesii*** (Genome Position: 15,329-17,171) | | | |
| 752-914 | 50 | 3.2 | TTTATGTTATTTTATTTTTGGGGTTATTTATTTTGAATATGTTCTTTTTG |
| 763-882 | 52 | 2.3 | TTTTTGGGGGGTTCTTTCTTATGAATAAGTTCCTTAGGTTTATGTTATTTCA |
| ***Diploria labyrinthiformis*** (Genome Position: 10,853-11,762) | | | |
| 657-715 | 15 | 3.7 | ATTCTTCTTCAATAG |
| ***Diploria labyrinthiformis*** (Genome Position: 16,105-16,905) | | | |
| 691-748 | 27 | 2.1 | TGTGGATTTATCATAATCAAATGCTTA |
| ***Oculina patagonica*** (Genome Position: 2,655-2,763) | | | |
| Ø | Ø | Ø | Ø |
| ***Stephanocoenia intersepta*** (Genome Position: 5,761-6,702) | | | |
| 508-558 | 23 | 2.3 | TGGTCTCAGGGGGTTTGCTCGGT |
| ***Stephanocoenia intersepta*** (Genome Position: 7,385-8,065) | | | |
| Ø | Ø | Ø | Ø |

**Supplementary Figure S1:** Minimum free energy predicted secondary structure of the long non-coding control regions of the four studied complete mitochondrial genomes of *Dichocoenia stokesii, Diploria labyrinthiformis, Oculina patagonica,* and *Stephanocoenia intersepta* from the web server RNAfold using default parameters (<http://rna.tbi.univie.ac.at/cgi-bin/RNAWebSuite/RNAfold.cgi> - Benson 1999).

| ***Dichocoenia stokesii*** (Genome Position: 15,329-17,171) | |
| --- | --- |
| 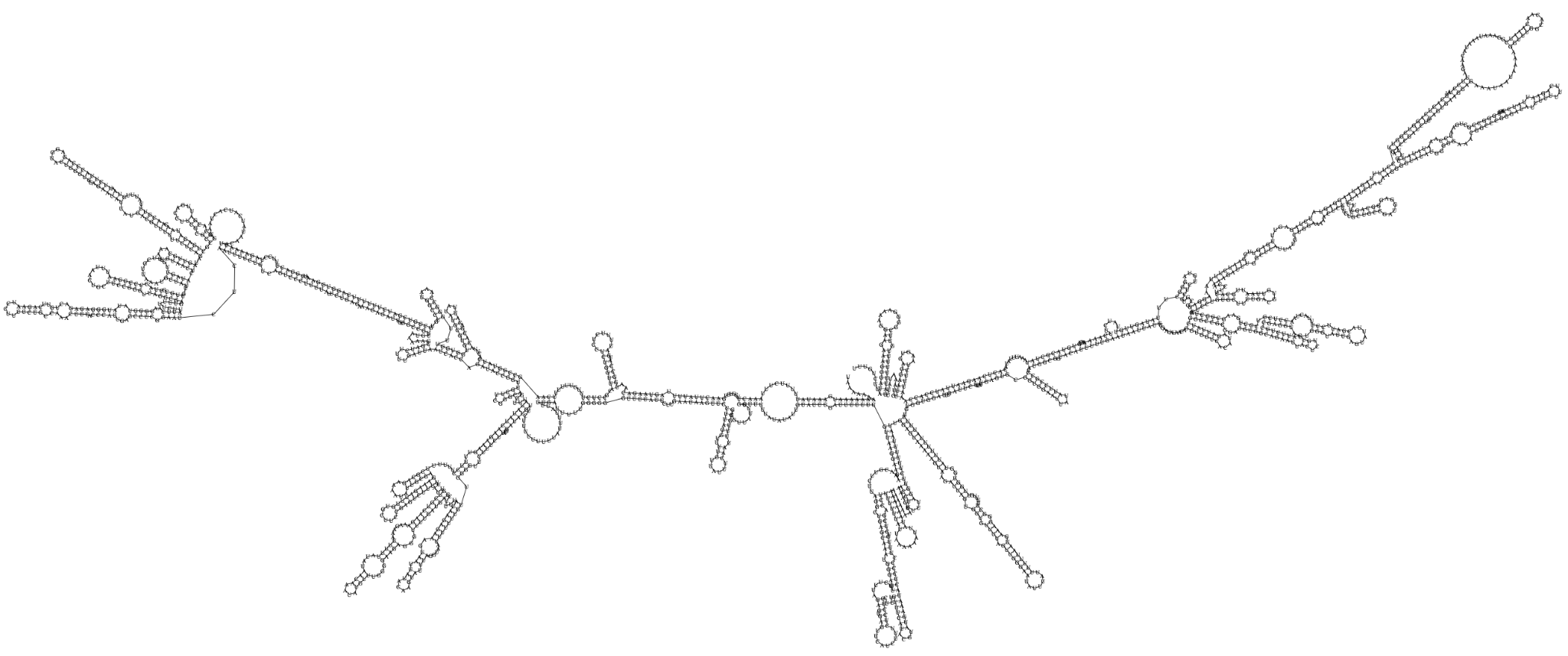 | |
| ***Diploria labyrinthiformis***  (Genome Position: 10,853-11,762) | ***Diploria labyrinthiformis***  (Genome Position: 16,105-16,905) |
| 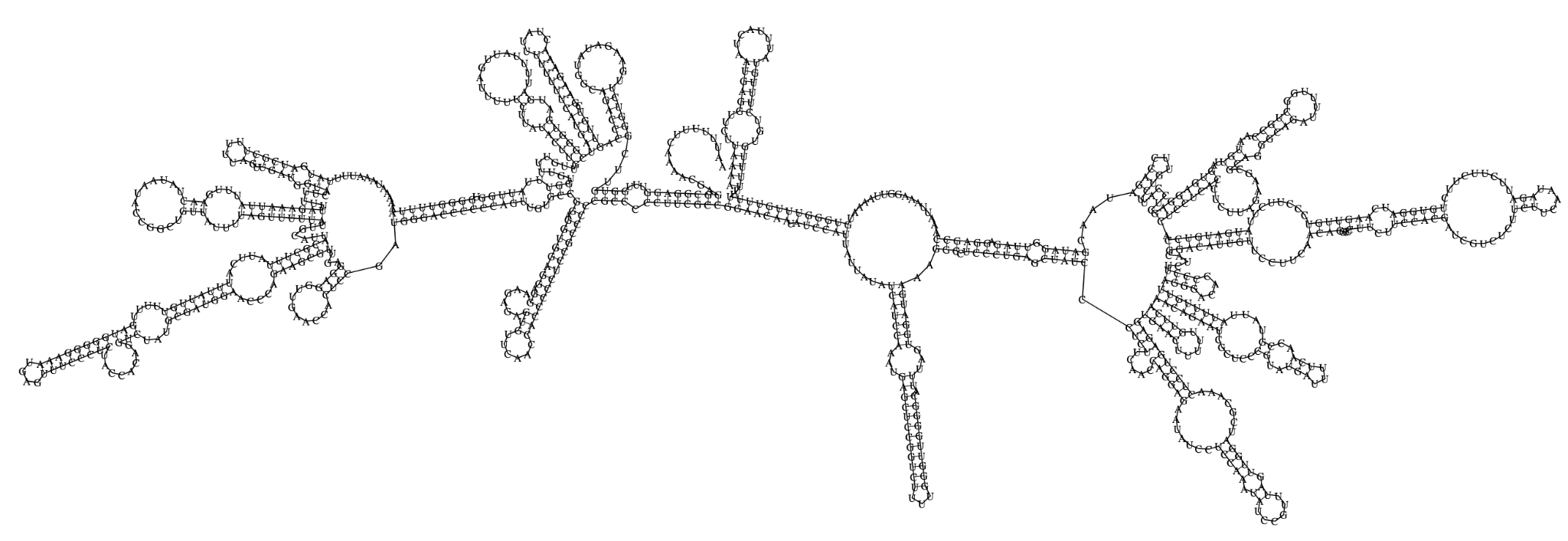 | 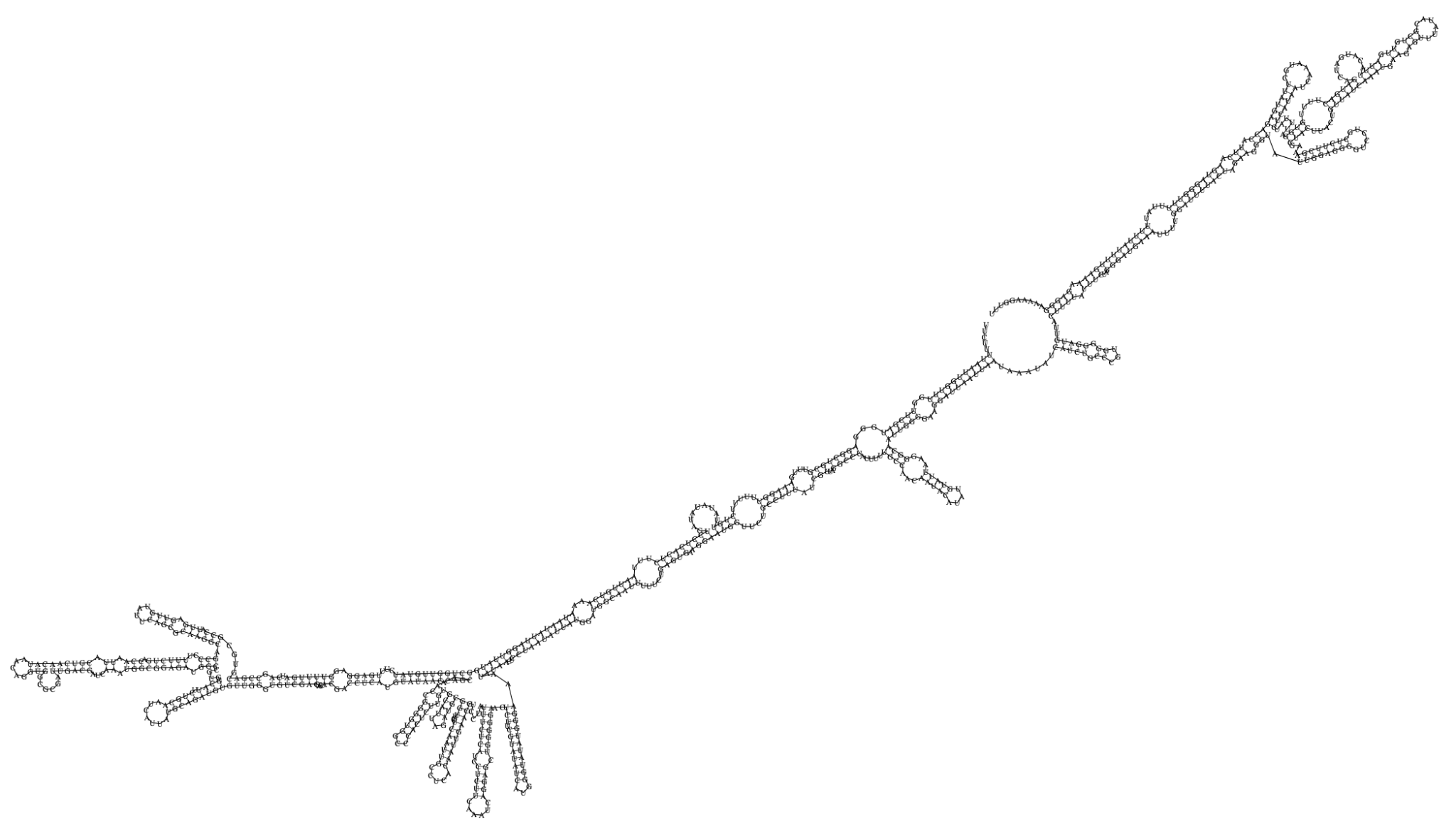 |
| ***Oculina patagonica*** (Genome Position: 2,655-2,763) | |
| 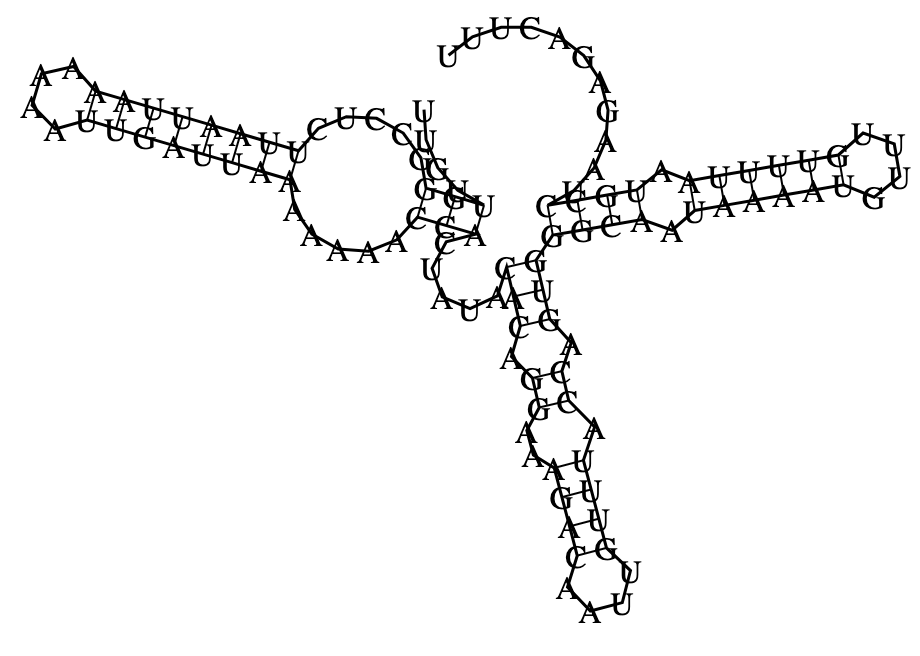 | |
| ***Stephanocoenia intersepta***  (Genome Position: 5,761-6,702) | ***Stephanocoenia intersepta***  (Genome Position: 7,385-8,065) |
| 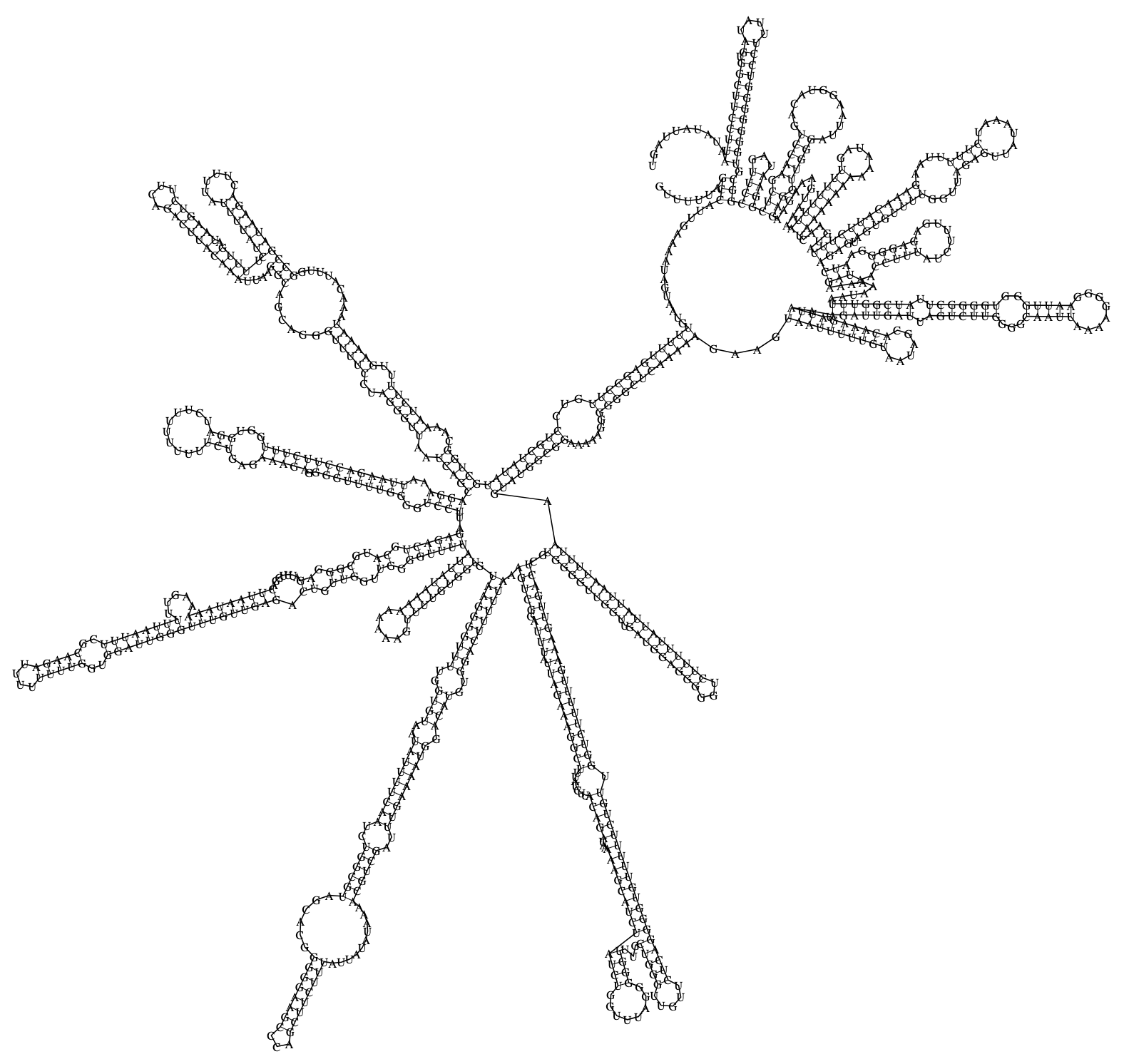 | 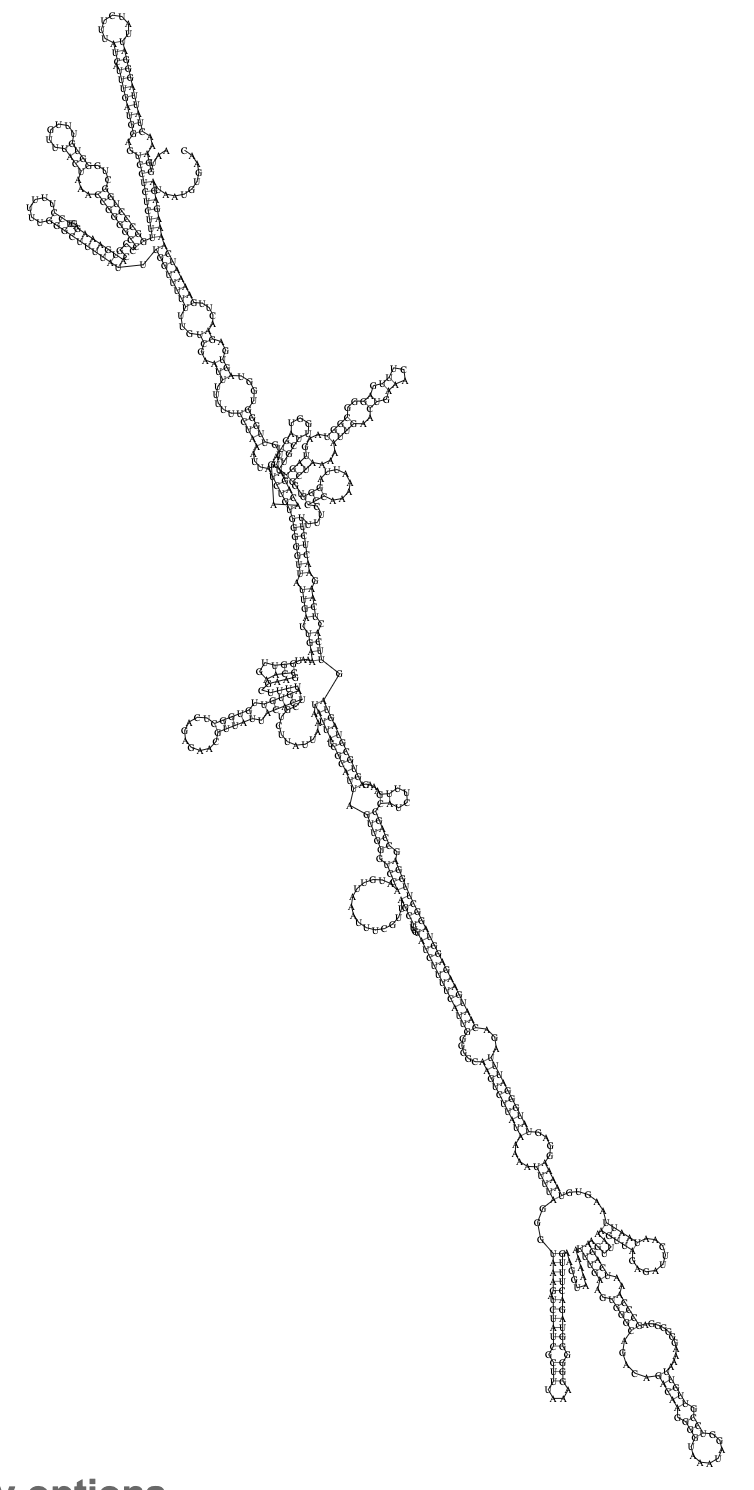 |
